# Melatonin Involved in Protective Effects against Cadmium Stress in *Wolffia arrhiza*

**DOI:** 10.3390/ijms24021178

**Published:** 2023-01-07

**Authors:** Magdalena Chmur, Andrzej Bajguz

**Affiliations:** Department of Biology and Plant Ecology, Faculty of Biology, University of Bialystok, Ciolkowskiego 1J, 15-245 Bialystok, Poland

**Keywords:** antioxidants, cadmium, duckweed, Lemnaceae, melatonin, primary metabolites, stress markers, *Wolffia*

## Abstract

Melatonin (MT) is a new plant hormone that protects against adverse environmental conditions. In the present study, the responses of *Wolffia arrhiza* exposed to cadmium (Cd) and MT were analyzed. Quantitative analysis of MT and precursors of its biosynthesis was performed using LC-MS-MS. The photosynthetic pigments and phytochelatins (PCs) contents were determined using HPLC, while protein and monosaccharides, stress markers, and antioxidant levels were determined using spectrophotometric methods. Interestingly, the endogenous level of MT and its substrates in *W. arrhiza* exposed to 1–100 µM Cd was significantly higher compared to the control. Additionally, the application of 25 µM MT and Cd intensified the biosynthesis of these compounds. The most stimulatory effect on the growth and content of pigments, protein, and sugars was observed in plants treated with 25 µM MT. In contrast, Cd treatment caused a decrease in plant weight and level of these compounds, while the application of 25 µM MT mitigated the inhibitory effect of Cd. Additionally, Cd enhanced the level of stress markers; simultaneously, MT reduced their content in duckweed exposed to Cd. In plants treated with Cd, PC levels were increased by Cd treatment and by 25 µM MT. These results confirmed that MT mitigated the adverse effect of Cd. Furthermore, MT presence was reported for the first time in *W. arrhiza*. In summary, MT is an essential phytohormone for plant growth and development, especially during heavy metal stress.

## 1. Introduction

Melatonin (MT; *N*-acetyl-5-methoxytryptamine) is a widely distributed signal molecule that regulates numerous physiological processes in animal and plant kingdoms. In humans and other animals, this is a neuroendocrine hormone secreted primarily by the pineal gland of the brain. In mammals, MT regulates circadian rhythm through the initiation of sleep and also affects concentration and alertness. Exogenously applied MT treats sleep disorders, for example, jet lag or delayed sleep phase syndrome [1]. Moreover, MT is an effective antioxidant in the treatment of certain neoplastic changes and has a favorable action against disorders of the nervous system [2]. In amphibians, MT lightens the skin color [3]. In plants, MT was detected in 1995 when its presence was confirmed in several species of edible plants [4,5]. The first MT receptor, CAND2/PMTR1, was detected in *Arabidopsis thaliana* in 2018. This receptor regulates stomatal closure through hydrogen peroxide (H_2_O_2_) and Ca^2+^ signals [6]. Since the discovery of this receptor, MT has been considered a new plant hormone. In both animals and plants, MT is synthesized from tryptophan (Trp) in a four-step pathway with serotonin (5HT) as the primary intermediate [7]. In recent years, the presence of MT has been confirmed in various species of plants [8]. Many studies demonstrate the regulatory functions of MT in plant growth and development, as well as in environmental stress response [9,10,11]. MT improves photosynthesis efficiency, the activity of Rubisco, and the stomatal conductance that improves the yield of seeds and fruits. Under adverse conditions, it regulates the metabolism of lipids, carbohydrates, and nitrogen compounds [12,13] and induces the biosynthesis of anthocyanins, carotenoids, and flavonoids. Moreover, MT plays a protective role against biotic and abiotic stresses such as cold, heat, salinity, drought, and heavy metals [9,14,15]. MT can enhance the synthesis of other compounds involved in heavy metal detoxification, such as glutathione (GSH), phytochelatins (PCs), or antioxidant enzymes. Moreover, MT acts as a direct scavenger of reactive oxygen species (ROS) [16]. Several studies demonstrate the positive role of MT in response to Cd stress [17,18]. For example, exogenous MT mitigates Cd toxicity by increasing PC and antioxidant levels in tomatoes [19].

Heavy metal contamination has become one of the main environmental problems worldwide. Cadmium (Cd) is one of the most harmful elements for all organisms. The toxic effect of Cd results from the ability to create covalent and ionic bonds with biogenic macro- and microelements, which affects their biological functions [20]. In plants, the early effect of Cd exposition is the inhibition of the overall growth of the organism. In addition, exposure to Cd negatively affects several physiological and biochemical processes that lead to the reduction or complete inhibition of photosynthesis, transpiration, and cellular respiration. Exposure of plants to high concentrations of Cd also contributes to a decrease in antioxidant enzyme activities that leads to the induction of oxidative stress and the accumulation of ROS [21,22]. ROSs that accumulate in plant cells react with proteins, lipids, and cell pigments, altering their functions that induce early aging and death of the plant [23]. A common negative effect of ROS activity is lipid peroxidation leading to the formation of aldehyde that disturbs the integrity of the cell membrane. Malondialdehyde (MDA), as a product of lipid peroxidation, indicates a mutagenic effect, and its level increases proportionally to the degree of ROS contamination in plants. Defense mechanisms against heavy metal damage are activated in the presence of metal [24].

There is little evidence on the presence and role of MT in aquatic plants [25,26,27]. *Wolffia arrhiza* (L.) Horkel ex Wimm. is one of these plants with strongly reduced organs, deprived of the stem, leaves, and root systems. At the same time, this is the smallest vascular plant, and its body size reaches about 1 mm in diameter. Flowering occurs rarely, and it often reproduces vegetatively [28,29]. Despite the simplified structure, *W. arrhiza* can live in freshwater and in organic-rich water and can change its feeding from photoautotrophic to heterotrophic [30]. Furthermore, *W. arrhiza* plays an important role in the absorption ability of heavy metals from polluted water. Thus, the fast reproduction and the ability to accumulate trace metals make *W. arrhiza* a good model for scientific analysis and practical uses for water purification [31].

Therefore, the main objective of the present work was to determine the endogenous level of MT and its intermediates in *W. arrhiza* treated with Cd and/or MT. Furthermore, the plant responses to the treatment with Cd and MT were also analyzed. Liquid chromatography coupled with mass spectrometry (LC-MS-MS) with multiple reaction monitoring (MRM) systems was used to identify MT and its intermediates. The use of LC-MS excludes interference with indoleacetic acid, which is also synthesized from Trp. The endogenous level of photosynthetic pigments and PCs was determined using the high-performance liquid chromatography (HPLC) method. Therefore, we hypothesize that (1) endogenous levels of MT and its intermediates increase under the influence of Cd and MT; (2) exogenously applied MT can alleviate the inhibitory effect of Cd on the growth rate and content of primary metabolites, i.e., photosynthetic pigments, monosaccharides, and proteins in *W. arrhiza*; (3) exogenous MT can increase the activity of enzymatic antioxidants, i.e., catalase (CAT), glutathione reductase (GR), ascorbate peroxidase (APX), and superoxide dismutase (SOD), also contributing to the increase in GSH and PCs content in plants exposed to Cd; (4) treatment with MT can reduce the content of stress markers, i.e., H_2_O_2_ and MDA in *W. arrhiza* exposed to Cd; and (5) the influence of Cd and MT on *W. arrhiza* is various in a concentration-dependent manner.

## 2. Results

### 2.1. Melatonin Improves the Growth Rate in W. arrhiza Exposed to Cadmium

**Figure 1:**
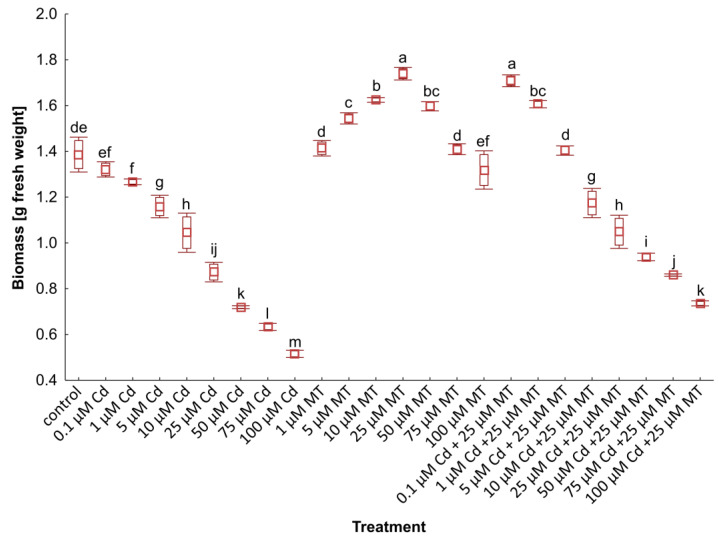
The biomass of *W. arrhiza* exposed to cadmium (Cd) or/with melatonin (MT) after 7 days of cultivation. The square represents the mean (*n* = 5, biological replicate). The lower and upper hinges correspond to the lower and upper bounds of the 95% confidence interval of the means. The lower and upper whiskers extend from the hinge to the mean ± standard deviation. Means with the same letters are not significantly different (*p* ≥ 0.05) according to Tukey’s post hoc test.

The fluctuation of growth is the primary parameter that describes the response to treatment with Cd, MT, and the combination of Cd and MT (Figure 1). In each variant, the biomass on the first day of cultivation was 1 g. Therefore, after 7 days of breeding, the biomass of non-exposed duckweed increased to 1.37 g. Plant exposure to Cd caused a progressive decline in biomass, reaching the highest level, a 68% decrease in plants exposed to 100 µM Cd. The application of MT resulted in a considerable increase in *W. arrhiza* growth compared to the control group. The most stimulating effect was observed in duckweed treated with 25 µM MT. Treatment with 25 µM MT caused the rise in *W. arrhiza* weight up to 1.74 g, which meant a 27% increase over control and a 74% increase compared to the first day of breeding. Otherwise, exposure to 100 µM MT caused a slight 3% decrease in duckweed weight compared to untreated plants.

The inhibitory effect of Cd on the growth of *W. arrhiza* was mitigated through treatment with 25 µM MT. The combinations of 25 µM MT with various concentrations of Cd stimulated duckweed weight compared to the plant treated with Cd alone. The growth rate of the plant exposed to 25 µM MT with 0.1, 1, and 5 µM Cd was higher than the control. Furthermore, the mixtures of 25 µM MT with 25 and 50 µM Cd caused 20% and 28% increases in biomass with plants exposed to 25 and 50 µM Cd alone, respectively.

### 2.2. Melatonin Decreases the Cadmium Level of W. arrhiza

The intracellular Cd level in *W. arrhiza* treated with Cd and MT after 7 days of breeding is presented in Table 1. The amount of Cd absorbed by *W. arrhiza* increased proportionally with applied Cd concentration. The highest accumulation of metal ions was observed in plants exposed to 100 µM Cd, while the highest biosorption rate was observed in plants treated with 50 µM Cd. However, the application of 25 µM MT caused a 10–20% decrease in metal accumulation compared to duckweed treated with Cd alone. The 23% decline in metal absorption was observed in plants exposed to 25 µM MT with 5 µM Cd to 5 µM Cd alone. Furthermore, the content of Cd decreased in the nutrient medium after 7 days of cultivation compared to day 1. The amount of remaining Cd in a medium containing a mixture of Cd with MT was higher than in a variant with Cd alone, which is related to decreased accumulation of Cd by *W. arrhiza*, which was growing with the addition of MT.

### 2.3. Cadmium Enhances the Endogenous Level of Melatonin and Its Precursors in W. arrhiza

The endogenous level of MT and its intermediates in *W. arrhiza* treated with Cd and MT is presented in Table 2. The LC-MS/MS analysis confirmed the presence of MT and six substrates of its biosynthesis, that is Trp, 5-hydroxytryptophan (5HTP), 5HT, *N*-acetylserotonin (NAS), tryptamine (TAM), and 5-methoxytryptamine (5MT). In the controls, the highest contents of 5HT, Trp, and MT were observed (62.22 ng/g, 31.31 ng/g, and 12.93 ng/g, respectively). Thus, the influence of Cd with MT on the endogenous content of detected compounds was analyzed. Interestingly, the application of Cd led to a considerable enhancement of both MT and all its intermediate content. The most stimulatory effect was observed in plants treated with 50 µM Cd, in which the level of MT increased about 5-fold in relation to the untreated plants. The Trp content was almost 4-fold higher, while the level of 5HT was about 2-fold higher compared to the control group. Exogenously applied MT also increased the endogenous content of MT, which is related to the absorption of the hormone by *W. arrhiza*. In addition to MT, the levels of its substrates also increased in the presence of exogenous MT. For example, Trp increased by 27% compared to the control. However, this is a significantly lower rise than in plants exposed to all concentrations of Cd except 0.1 µM. However, the combination of 50 µM Cd with 25 µM MT caused the highest increase in the content of all detected compounds. The cumulative action of 50 µM Cd with 25 µM MT resulted in an 8.3% increase in Trp content and a 5% increase in 5HT content in relation to duckweed treated with 50 µM Cd alone.

### 2.4. Melatonin Increases the Content of Photosynthetic Pigments, Protein, and Monosaccharides in W. arrhiza Exposed to Cadmium

**Figure 2:**
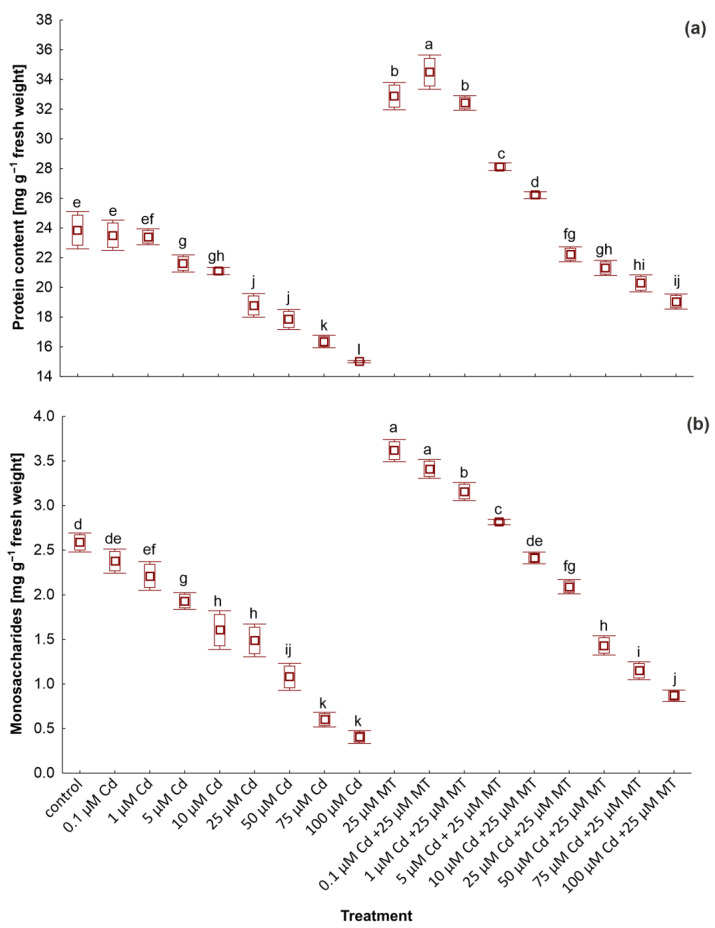
The content of soluble proteins (**a**) and monosaccharides (**b**) in *W. arrhiza* exposed to cadmium (Cd) or/with melatonin (MT) after 7 days of cultivation. The square represents the mean (*n* = 5, biological replicate). The lower and upper hinges correspond to the lower and upper bounds of the 95% confidence interval of the means. The lower and upper whiskers extend from the hinge to the mean ± standard deviation. Means with the same letters are not significantly different (*p* ≥ 0.05) according to Tukey’s post hoc test.

The application of Cd and MT had various effects on plant metabolism. Treatment with Cd caused a decrease in the content of primary metabolites, i.e., photosynthetic pigments (Table 3), soluble proteins, and monosaccharides (Figure 2). Plant exposure to 0.1 µM Cd resulted in an inconsiderable decrease in the level of pigments, protein, and sugars. However, treatment with 100 µM Cd caused the most inhibitory effect on the content of all the above metabolites. For instance, the endogenous level of chlorophyll *a*, α-carotene, and cryptoxanthin decreased by 60%, 54%, and 38%, respectively, in relation to plants untreated with Cd. An opposite effect was observed in duckweed exposed to the hormone. The treatment with 25 µM MT caused a significant enhancement of the content of the pigment compared to the control. The amount of protein and sugars also increased in plants with the addition of 25 µM MT. Furthermore, the application of 25 µM MT mitigated the inhibitory effect of Cd on the content of primary metabolites. The level of chlorophyll *a*, monosaccharides, and proteins increased by 44%, 43%, and 34%, respectively, in duckweed treated with a mixture of 25 µM MT and 100 µM Cd compared to plants treated with 100 µM Cd alone. The combination of the hormone with other concentrations of metal had similar results. Furthermore, the content of all detected pigments, except lutein, in plants exposed to a mixture of MT with 0.1, 1, and 5 µM Cd was considerably higher than in the control group. Similar results were obtained for sugars and proteins.

### 2.5. Melatonin Reduces the Content of Malondialdehyde and H_2_O_2_ in W. arrhiza Treated with Cadmium

**Figure 3:**
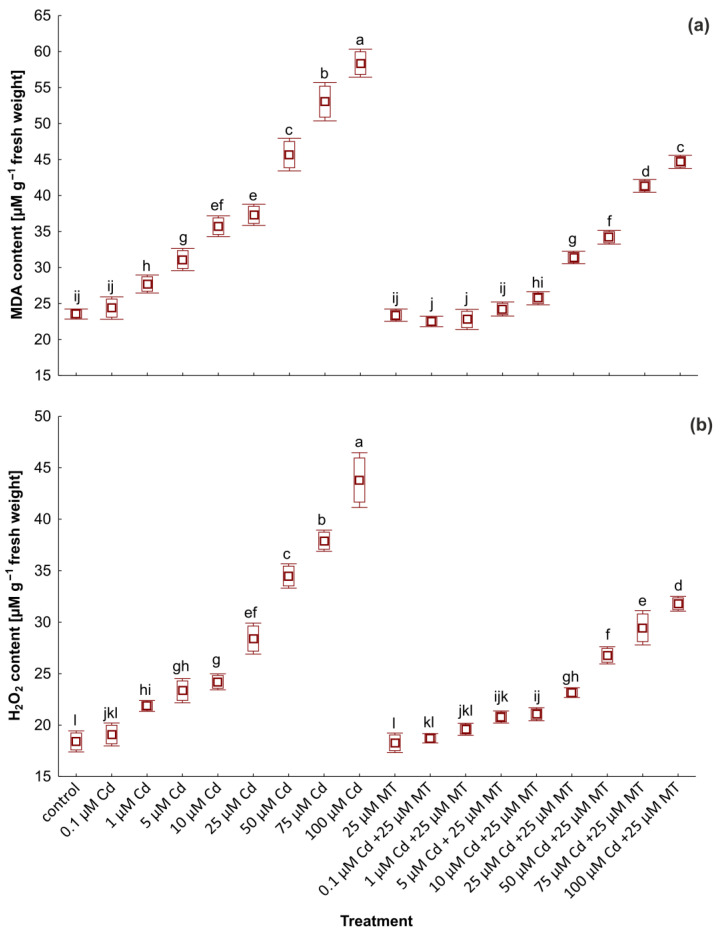
The content of malondialdehyde (MDA) (**a**) and hydrogen peroxide (H_2_O_2_) (**b**) in *W. arrhiza* exposed to cadmium (Cd) or/with melatonin (MT) after 7 days of cultivation. The square represents the mean (*n* = 5, biological replicate). The lower and upper hinges correspond to the lower and upper bounds of the 95% confidence interval of the means. The lower and upper whiskers extend from the hinge to the mean ± standard deviation. Means with the same letters are not significantly different (*p* ≥ 0.05) according to Tukey’s post hoc test.

The intensified lipid peroxidation process and the excessive synthesis of H_2_O_2_ are essential indicators of oxidative stress in plants exposed to heavy metals. The level of MDA and H_2_O_2_ in plants treated with Cd and MT is shown in Figure 3. Cd stress caused considerable increases in MDA and H_2_O_2_ levels in *W. arrhiza* cells, reaching the highest value in plants treated with 100 µM Cd. Treatment with MT alone did not affect the endogenous level of stress markers compared to the control plant. However, after MT application in plants treated with Cd, a remarkable decrease in the content of stress markers was reported. In plants exposed to a mixture of 100 µM Cd with 25 µM MT, MDA and H_2_O_2_ levels were reduced by approximately 24% and 28%, respectively, compared to the group without hormone addition. The content of MDA and H_2_O_2_ in plants treated with a mixture of MT with 1 µM Cd was slightly lower than in the control group, although, in duckweed exposed to 1 µM Cd alone, the content of these compounds was higher than the control by 17% and 19%, respectively.

### 2.6. Melatonin Stimulates the Content of Glutathione and Phytochelatins in W. arrhiza Treated with Cadmium

**Figure 4:**
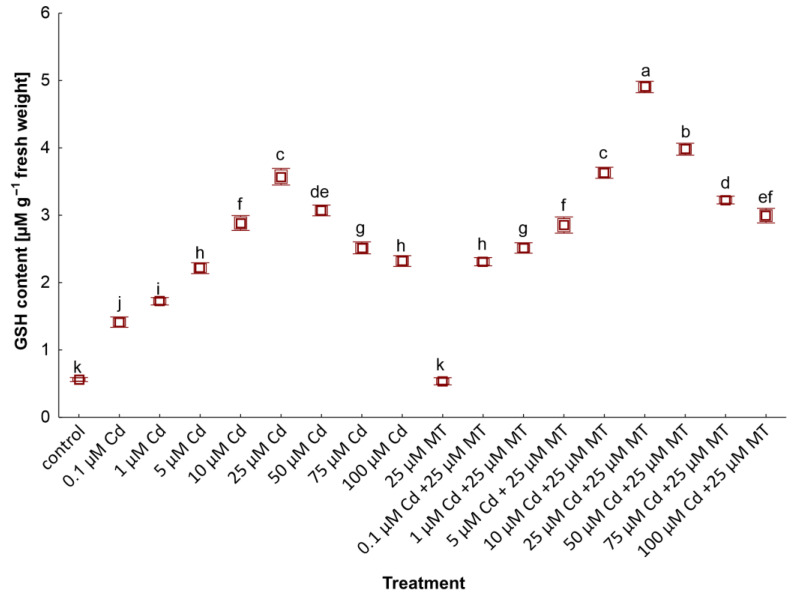
The content of glutathione (GSH) in *W. arrhiza* exposed to cadmium (Cd) or/with melatonin (MT) after 7 days of cultivation. The square represents the mean (*n* = 5, biological replicate). The lower and upper hinges correspond to the lower and upper bounds of the 95% confidence interval of the means. The lower and upper whiskers extend from the hinge to the mean ± standard deviation. Means with the same letters are not significantly different (*p* ≥ 0.05) according to Tukey’s post hoc test.

**Figure 5:**
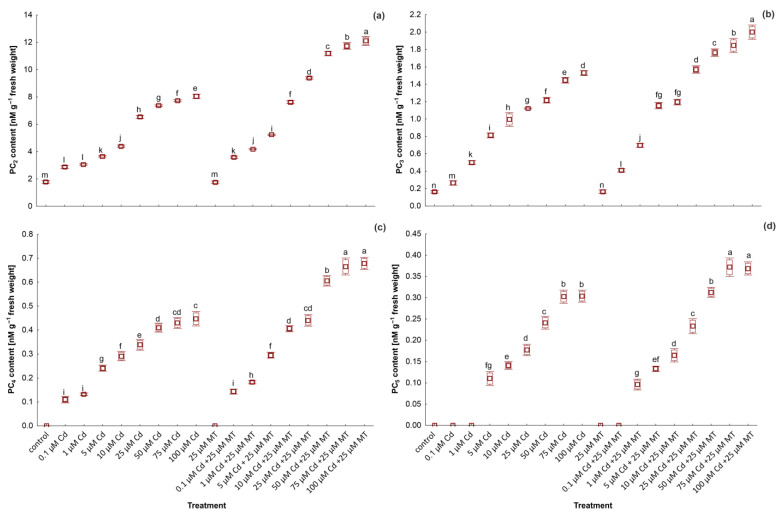
The content of phytochelatins (PC_x_): PC_2_ (**a**), PC_3_ (**b**), PC_4_ (**c**), and PC_5_ (**d**) in *W. arrhiza* exposed to Cd or/with MT after 7 days of cultivation. The square represents the mean (*n* = 5, biological replicate). The lower and upper hinges correspond to the lower and upper bounds of the 95% confidence interval of the means. The lower and upper whiskers extend from the hinge to the mean ± standard deviation. Means with the same letters are not significantly different (*p* ≥ 0.05) according to Tukey’s post hoc test.

The content of GSH and PC_2-5_ in *W. arrhiza* treated with Cd and MT was presented in Figure 4 and Figure 5, respectively. In the controls, GSH and a slight amount of PC_2-3_ were detected. In plants treated with Cd, PC synthesis significantly increased, reaching the highest value under 100 µM Cd. The highest amount of GSH was observed in plants treated with 25 µM Cd. Among all identified PCs, PC_2_ dominated, while PC_3-5_ was found in a lower amount. The application of 25 µM MT alone did not increase thiol compound synthesis. However, GSH and PC_2-5_ were increased by 29%, 50%, 31%, 52%, and 33%, respectively, in the plant after 25 μM MT treatment under 100 µM Cd stress compared to plants exposed to 100 µM Cd alone. Furthermore, PC_5_ was reported only in plants treated with 1 µM and MT. The total PC amounts in plants treated with 100 µM Cd and 25 µM MT mixture was 15 nM/g FW, almost 50% higher than in plants exposed to Cd without hormone addition. 

### 2.7. Melatonin Intensifies the Antioxidants Activity in W. arrhiza Treated with Cadmium

**Figure 6:**
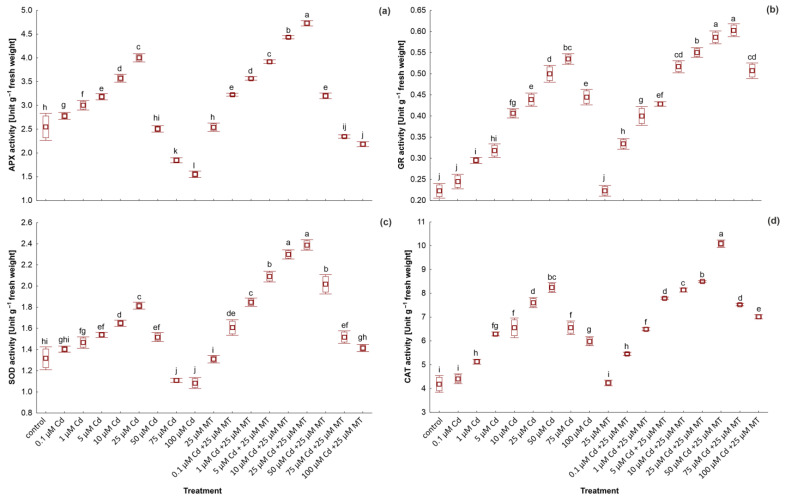
The activity of ascorbate peroxidase (APX) (**a**), glutathione reductase (GR) (**b**), superoxide dismutase (SOD) (**c**), and catalase (CAT) (**d**) in *W. arrhiza* exposed to cadmium (Cd) and/or melatonin (MT) after 7 days of cultivation. The square represents the mean (*n* = 5, biological replicate). The lower and upper hinges correspond to the lower and upper bounds of the 95% confidence interval of the means. The lower and upper whiskers extend from the hinge to the mean ± standard deviation. Means with the same letters are not significantly different (*p* ≥ 0.05) according to Tukey’s post hoc test.

The enhanced synthesis of antioxidant enzymes is an essential parameter describing plants’ responses to various types of stress. The activity of APX, CAT, GR, and SOD in *W. arrhiza* treated with Cd and MT is presented in Figure 6. Generally, antioxidant activity increased under the influence of all Cd concentrations compared to the unstressed group, except APX and SOD in plants exposed to 75 µM and 100 µM Cd. The highest activity of APX and SOD was observed in *W. arrhiza* exposed to 25 µM Cd and GR in plants treated with 75 µM MT, while CAT was the highest in duckweed exposed to 50 µM Cd. Treatment with MT alone had no impact on antioxidant activity. However, MT greatly stimulated the synthesis of antioxidants in plants exposed to various doses of Cd. For example, the activity of CAT in plants growing with the addition of 100 µM Cd and 25 µM MT was 17% higher than in plants grown without hormone addition. However, the greatest increase in CAT activity was observed under the influence of MT in plants treated with 10 µM Cd (24% increases compared to plants treated with Cd alone).

## 3. Discussion

The toxic effect of Cd on plant growth and development is obvious. Poisoning with Cd has adverse effects on plant conditions, where exposure to Cd causes a deleterious effect on plants, inhibits growth, and decreases the content of proteins and chlorophylls, as well as enhances the level of H_2_O_2_ and MDA [32,33]. Although the influence of Cd on biochemical processes was analyzed in a variety of plant species, the impact of MT is quite a new issue, especially in water plants. *W. arrhiza,* an aquatic plant without roots, has an absorption ability of chemical compounds from the water environment [31]. Therefore, the absorption rate of Cd ions from Hutner’s medium was notable. The present results revealed a positive correlation between the concentration of Cd added to the medium and its intracellular level in *W. arrhiza* cells after 7 days of cultivation. However, Cd accumulation decreased after 25 µM MT application, which is related to an increase in PC_2-5_. MT can also reduce the accumulation of Cd through the expression of genes responsible for the transport that leads to lower entering of Cd inside plant cells [34]. Reduced Cd uptake in *W. arrhiza* is probably caused by MT’s ability to modulate the activity of the ATP-binding cassette transporter, a Cd extrusion pump [35]. Reduced intracellular Cd content under the influence of MT was also observed, for example, in *Brassica napus* seedlings [36] or roots and leaves of radish plants [34]. The foliar spray of MT also significantly decreased the accumulation of Cd in rice leaves [37].

The main aim of the present work was to determine the endogenous level of MT and its substrates in *W. arrhiza* treated with various concentrations of Cd ions. Since 2010, studies concerning the presence and activity of MT in plants have been very well known. During this time, the endogenous level of MT was confirmed in various plant species, including agricultural and wild types, and plant organs, i.e., seeds, roots, stems, leaves, flowers, and fruits. Several methods are used to determine the endogenous level of MT and its precursors in plants. The most popular technique is HPLC, using fluorescence or electrochemical detectors. However, due to the low concentration of these compounds, more specific and sensitive methods are required. Therefore, the LC-MS-MS technique is an excellent tool for quantifying MT and its intermediates in different species of plants [38]. For example, in 2012, the occurrence of MT and 5HT was confirmed in the green and roasted beans of *Coffea canephora* and *Coffea arabica* [39]. Moreover, the MT biosynthesis pathway has been widely described, mainly in *Arabidopsis thaliana* and *Oryza sativa* plants [40]. Thus, the initial compound in the biosynthesis of MT is localized in chloroplast amino acid Trp, which is converted to 5HTP by tryptophan 5-hydroxylase; then, 5HTP is changed to 5HT, and this reaction is catalyzed by tryptophan decarboxylase. 5HT can also be synthesized through the decarboxylation of Trp to TAM by tryptophan decarboxylase and then through the hydroxylation of TAM to 5HT by tryptamine-5-hydroxylase. Next, 5HT is changed in NAS by serotonin-*N*-acetyltransferase, and finally, NAS is converted to MT by NAS methyltransferase, which is localized in the cytosol. 5HT can also be changed into 5MT by hydroxy-indole-*O*-methyltransferase and is finally transformed into MT through the serotonin-*N*-acetyltransferase localized in the chloroplast [41]. With regard to the previous reports, there is little evidence about the identification of MT intermediates. In addition to MT, the occurrence of Trp, 5HTP, TAM, 5HT, and NAS was confirmed in rice seeds [42]. In turn, the MT, Trp, TAM, and 5HT level was measured in tomato plants [43]. In the present study, MT and six of its intermediates were detected. This detection suggests that MT is synthesized both in chloroplasts and cytosols of *W. arrhiza* cells [36]. Furthermore, the present study indicated that the presence of Cd significantly stimulated the synthesis of MT and its precursors in the *W. arrhiza* plant. Melatonin and its substrate levels considerably increased in response to Cd treatment. Exposition on 50 µM Cd caused the highest synthesis of MT in *W. arrhiza* after 7 days of cultivation. These results can confirm the antioxidant activity of MT [44]. Recent research by Lee et al. [45] also indicated the stimulating effect of Cd on MT, NAS, and 5HT in rice seedlings. Other studies [46] demonstrated the elevated level of MT in rice leaves treated with Cd. In summary, MT is the next phytohormone that was indicated in *W. arrhiza.* The previous report confirmed the presence of abscisic acid, gibberellic acid, brassinosteroids, and cytokinins in this duckweed [47,48].

In addition to hormone studies, the growth and metabolism under the influence of Cd and MT were also analyzed. The first immediately visible effect of the action of Cd is inhibited multiplication process and the reduced biomass of *W. arrhiza* cultures. In the current study, Cd concentrations higher than 10 µM not only led to a reduction in the growth but also induced decay of *W. arrhiza* cultures. Similar to growth inhibition, Cd-induced damage in plant cells was also assayed by decreasing the content of primary metabolites, including photosynthetic pigments, monosaccharides, and proteins. Chlorophylls are involved in the absorption of light from solar power for ATP production, and analysis of their amount can determine the photosynthesis rate in plants. Carotenoids, as the second group of photosynthetic pigments, have antioxidant activity and participate in the protection of chlorophylls against photo-oxidative destruction. Carotenoids are divided into carotenes and oxygen-rich and oxygen-poor xanthophylls [48,49]. Photosynthetic pigments are necessary for the light phase of the photosynthesis process. The influence of heavy metals on chlorophylls and carotenoids with separated xanthophyll content was studied in algae and agricultural uses. Similar to our results, Pb caused a decrease in pigment amount in *Acutodesmus obliquus* [50,51]. In the current work, the reduced level of pigments was observed under the influence of Cd. The toxic effect of Cd on photosynthesis can be caused by the ability to bind to crucial elements of photosynthetic pigments, including Mg^2+^, Ca^2+^, Fe^2+^, and K^+^, resulting in chlorophyll degradation and chlorosis [52]. The reduced pigment content in *W. arrhiza* treated with Cd could also be due to the peroxidation of chloroplast membranes caused by elevated ROS synthesis [53]. The reduced level of monosaccharides is related to the degradation of photosynthesis pigments. Sugars, mainly glucose, are synthesized during photosynthesis and constitute an important reservoir of energy essential for all biochemical processes in plants [54]. Cd can directly degrade the protein, but a decreased level of proteins is also connected with DNA damage caused by heavy metals [55]. In opposition to Cd, the exposure to MT caused a considerable increase in both growth rate and primary metabolite content. In this study, MT at a concentration of 25 µM had the most stimulating effect on the growth of *W. arrhiza*. Regarding the literature data, optimal MT concentrations are various and depend on plant species. For example, the highest growth of *Stevia rebaudiana* seedlings was obtained after treatment with 20 µM MT [56]. Moreover, the application of MT significantly mitigated the phytotoxicity of Cd in *W. arrhiza*. Exogenous treatment with MT alleviated the inhibitory effect of Cd on the growth and the primary metabolites in *W. arrhiza*. The direct role of MT in the preservation of chlorophyll content is the downregulation of the expression of pheide an oxygenase (PAO) and chlorophyllase (CLH1) enzymes involved in chlorophyll degradation [57]. According to previous reports, exogenously applied MT alleviated the inhibitory effect of 100 µM Cd on the chlorophyll content and the net photosynthetic rate in *Nicotiana tabacum* leaves [32]. In another study, MT increased chlorophyll content and photosynthetic rate in *Brassica napus* seedlings treated with Cd [36]. The positive role of 15 µM and 50 µM MT on the growth and level of soluble proteins and photosynthetic pigments was also obtained for the *Malva parviflora* [58]. In turn, the stimulative effect of MT on the growth and level of primary metabolites was observed in maize seedlings growing in drought-stress conditions [59].

The Cd-caused oxidative stress in *W. arrhiza* was manifested by elevated production of ROS that contributed to the intensified lipid peroxidation and synthesis of MDA. Furthermore, the activity of antioxidants was increased in plants exposed to Cd stress. Antioxidant enzymes are responsible for the alleviation of ROS-induced detrimental effects. SOD is an enzyme that catalyzes the conversion of the O_2_ radical to H_2_O_2_, which is decomposed into O_2_ and H_2_O through the CAT. APX, a key enzyme in the ascorbate-glutathione cycle, catalyzes the reduction of H_2_O_2_ to H_2_O by utilizing ascorbate as a substrate [32]. In turn, GR plays an important role in reducing GSSG to GSH, and increasing its activity is related to elevated ROS production during abiotic stresses [60]. In the current work, the activities of CAT, APX, GR, and SOD were significantly enhanced in the plant exposed to Cd in relation to the control. The increase in CAT and APX is related to the increased H_2_O_2_ level in Cd-treated plants. MT has positive effects on active ROS scavenging, antioxidant capacity, biosynthesis of sulfhydryl compounds, and the sequestration of Cd in vacuoles [61]. In this study, exogenous MT improved the antioxidant systems responsible for ROS scavenging. The cumulative effect of 25 µM MT with Cd intensified the activity of these enzymes, although the influence of MT alone on the enzyme activities was not observed. Simultaneously, the enhanced stress markers were alleviated after MT treatment, which is consistent with studies in safflower seedlings [60]. These results indicated the protective role of MT against lipid peroxidation [62,63]. The previous analysis showed the stimulative effect of MT on the activity of antioxidant enzymes in *Triticum aestivum* [64], *Catharanthus roseus* [65], *Fragaria ananassa* [66], or *Raphanus sativus* [34] exposed to the Cd. The present results showed that MT contributes to the mitigation of Cd-induced stress and confirmed the antioxidant capacity of MT under abiotic stress conditions.

PCs play an essential role in the detoxification of Cd ions through their chelation in the plant cytosol and accumulation in the vacuole as high molecular weight complexes. Transport of Cd ions to the vacuolar space is of importance because the toxic effect of sequestered in vacuole metal is less destructive. The synthesis of PCs is greatly induced by the presence of Cd in the cytosols of cells. GSH, as a substrate, is primarily deprived of glycine, resulting in the formation of γ-glutamylcysteine (γ-Glu-Cys) molecule that is transferred to another GSH molecule, creating PC_2_. γ-Glu-Cys can also be attached to another PC acceptor molecule for the synthesis of PC_3_, PC_4_, or PC_5_ [67]. In the presence of metal ions, the first and the most stable type of PC is PC_2_, while its amount is the most abundant [68]. The current work shows that Cd application significantly stimulated PC_2-5_ biosynthesis in *W. arrhiza* cultures. While in untreated plants, the presence of PC_2-3_ is probably caused by the presence of different chemical elements in Hutner’s medium, and these could induce the synthesis of PCs. Under the influence of high Cd concentrations, the level of GSH was lower than at concentrations up to 25 µM, probably as a result of the utilization of GSH for PC synthesis [69]. These results agree with Bellini et al. [70], who showed a lower amount of GSH in the *Leptodictyum riparium* exposed to 360 µM Cd compared to 36 µM Cd, with a simultaneously elevated level of PC_2-4_ in plants treated with a higher dose of Cd. Then, the present study indicated that the synthesis of GSH and PCs under Cd stress in *W. arrhiza* cultures was further intensified by exogenous MT treatment. MT effectively induces PC biosynthesis in *W. arrhiza* stressed with Cd, resulting in higher binding of Cd to PC. Hasan et al. [19] showed that the accumulation of PCs in the roots of *Solanum lycopersicum* treated with Cd was increased under the influence of MT. Similar results were reported for *Carthamus tinctorius* seedlings [60]. In summary, MT-induced production of PCs and storage of Cd-PC complexes in vacuoles may play a crucial role in conferring Cd tolerance in plants [71].

In summary, the presence of MT and its intermediates (i.e., Trp, TAM, 5HTP, 5HT, NAS, and 5MT) was reported for the first time in *W. arrhiza*, as well as the influence of Cd and MT on their content. The level of these metabolites increased significantly in *W. arrhiza* exposed to both Cd and MT. Furthermore, the effect of exogenously applied Cd and MT on the *W. arrhiza* growth and development was also analyzed. Treatment with Cd caused a decrease in plant growth and the content of primary metabolites, such as soluble protein, monosaccharides, and photosynthetic pigments, in a dose-dependent manner. In turn, the exposure to Cd increased the accumulation of PCs and stress markers and enhanced the synthesis of antioxidants. The addition of 25 µM MT mitigated the inhibitory effect of Cd on the growth and metabolites content, declining MDA and H_2_O_2_ amount in plants treated with Cd, and MT treatment intensified the presence of Cd ions. In plants treated with MT, the accumulation of Cd was lower, which is related to increased PC content. Therefore, MT limits Cd stress in the *W. arrhiza* cultures.

## 4. Materials and Methods

### 4.1. Plant Material and Growth Conditions

The culture of *W. arrhiza* was received from the Faculty of Biology of the University of Bialystok. The breeding duckweed was grown under optimal conditions at 22.0 ± 0.5 °C, a 16 h photoperiod (photon flux of 100 µmol/m/s), and a relative humidity of 65%. One gram of plant was placed in a sterile glass vessel containing 200 mL of Hutner medium with the following composition: 500 mg/L ethylenediaminetetraacetic acid (EDTA), 500 mg/L MgSO_4_·7H_2_O, 400 mg/L KH_2_PO_4_, 354 mg/L Ca(NO_3_)_2_·4H_2_O, 200 mg/L KOH, 200 mg/L NH_4_VO_3_, 65.9 mg/L ZnSO_4_·7H_2_O, 25.2 mg/L Na_2_Mo_4_·2H_2_O, 24.9 mg/L FeSO_4_·7H_2_O, 17.9 mg/L MnCl_2_·4H_2_O, 14.2 mg/L H_3_BO_3_, 3.95 mg/L CuSO_4_·5H_2_O, and 0.2 mg/L Co(NO_3_)_2_·6H_2_O [72]. The entire experiment was divided into two major stages. In the first step for duckweed, the cultures were exposed to Cd in concentrations of 0.1, 1, 5, 10, 25, 50, 75, and 100 µM, or MT in concentrations of 1, 5, 10, 25, 50, 75, and 100 µM. The initial solution of the metal was prepared by dissolution of CdCl_2_ powder in distilled water, while MT was dissolved in 20% (*v*/*v*) ethanol (EtOH). The final amount of EtOH added to the medium did not influence the growth of *W. arrhiza.* The various solutions were prepared by diluting metal and MT in Hutner’s medium. After 7 days of exposure, the plant biomass was separated from Hutner’s medium by filtration using a vacuum pump (KNF Neuberger, Inc., Trenton, NJ, USA). The collected *W. arrhiza* cultures were then weighed (Precisa 180A, PAG Oerlikon AG, Zürich, Switzerland) and homogenized in liquid nitrogen using a mortar and pestle. The resulting powder was used in the subsequent analysis, excluding the measurement of the intracellular level of Cd, because this parameter was analyzed with the use of the fresh weight of the plant. In the second experiment, plant cultures were exposed to a mixture of 25 µM MT with Cd. The concentration of 25 µM MT was selected because it indicated the most stimulating effect on the growth and level of analyzed compounds in *W. arrhiza*. The subsequent parts of the sample preparation were analogs to step one. After 7 days of breeding, the selected biochemical parameters were analyzed. In addition to hormonal studies, analysis of growth and level of primary metabolites, as well as stress markers and antioxidants, is essential for research concentrated on plants because these parameters are a basic indicator of plant responses.

### 4.2. Chemicals

All chemicals for Hutner’s medium, CdCd_2_, MT, the standards of MT intermediates (i.e., Trp, 5HTP, TAM, 5HT, NAS, 5MT), Bradford and Somogyi reagents, bovine albumin standard, pyridine, acetone, chloroform, and GSH were purchased from Sigma-Aldrich (St. Louis, MO, USA). Methanol (MeOH), EtOH, acetonitrile (ACN), water, and formic acid (FA) were purchased from Merck KGaA (Darmstadt, Germany). Standards of photosynthesis pigments, i.e., chlorophyll *a*, chlorophyll *b*, α-carotene, β-carotene, astaxanthin, cryptoxanthin, neoxanthin, violaxanthin, zeaxanthin, and lutein, were purchased from DHI (Horsholm, Denmark). PC_2-5_ standards were purchased from AnaSpec (Fremont, CA, USA). Other chemicals and reagents were purchased from Sigma-Aldrich (St. Louis, MO, USA).

### 4.3. Determination of Cadmium Content

The intracellular concentration of Cd ions was measured by flame atomic absorption spectrometry (AAS) using a Solaar M6 (TJA Solutions, Cambridge, UK) spectrometer with a deuterium background correction system. The absorbance of Cd^2+^ was determined in an air–acetylene flame with a 0.5 nm spectral bandpass at 228.8 nm [73]. Thus, 1 g of *W. arrhiza* fresh weight was suspended in 20 mL of 100 µM Na_2_EDTA solution for 10 min to remove Cd ions from the plant’s external surface. The purified plants were dried in an oven for 12 h at 65 °C. The dry samples were suspended in 6 mL of 65% HNO_3_ (trace select purity) and heated in the microwave system for 15 min. Furthermore, on the 1st and 7th days of cultivation, 2 mL of Hutner’s medium was collected to determine the Cd content in the medium. The prepared samples were analyzed.

### 4.4. Quantitative Analysis of Melatonin and Its Intermediates

The endogenous level of MT and the substrates of its biosynthesis were determined using the method of Lee et al. [45]. For sample preparation, 0.5 g of *W. arrhiza* powder was placed in the 2 mL Eppendorf tube containing 1 mL of chloroform and homogenized in the bead mill (50 Hz, 10 min, TissueLyser LT; Qiagen GmbH, Düsseldorf, Germany). The obtained extract was shaken in a laboratory thermomixer (90 rpm, 22 °C, 60 min; Eppendorf Corporate, Hamburg, Germany) for hormone extraction. Then, the sample was evaporated to dryness using a centrifugal vacuum concentrator (45 °C, Labconco CentriVap Micro IR, Kansas City, MO, USA). The obtained pellet was dissolved in 100 μL of 40% MeOH and transferred to a glass vial with a 250 μL insert with polymer feet.

Identification and quantification of MT and its intermediates were performed using the Shimadzu LC-MS-MS-8050 system consisting of an autosampler, pump, degasser, column oven, and mass spectrometer with triple quadrupole (Shimadzu Corporation, Kyoto, Japan); 10 µL of each sample was injected on the Waters XSelect C_18_ column (250 mm × 3.0 mm, 5 μm). The temperature of the column oven was 30 °C. Mobile phase A was H_2_O with 0.01% (*v*/*v*) FA, and phase B was ACN with 0.01% (*v*/*v*) FA. The linear gradient of phase A was from 80% in the 1st min to 50% in the 10th min, 50% from the 10th min to the 15th min, and 80% after the 15th min. The analysis time was 15 min. The flow was 0.5 mL min^−1^. The chromatographic properties detected compounds in positive ionization scan mode are presented in Table 4. The chromatograms of the detected compounds are presented in Figure S1. Analytical data were analyzed using Shimadzu LabSolutions version 5.6 Software for LC/MS.

### 4.5. Determination of Photosynthetic Pigments

The photosynthetic pigment content in *W. arrhiza* was determined using high-performance liquid chromatography (HPLC) according to the method by Zapata et al. [74]. Therefore, 0.5 g of plant powder was suspended in 99.9% (*v*/*v*) MeOH and homogenized in a bead mill (50 Hz, 10 min; TissueLyser LT, Qiagen, Germany) to disrupt cells. The resulting homogenate was left in the refrigerator for 12 h for pigment isolation. The samples were centrifuged (2800× *g*, 10 min; MPW-55 Med. Instruments, Gliwice, Poland), and the obtained extract was analyzed.

For pigment separation and analysis, the Agilent 1260 Infinity Series HPLC apparatus (Agilent Technologies, Inc., Santa Clara, CA, USA) with quaternary pump with inline vacuum degasser, refrigerated autosampler with autoinjector sample loop, thermostatic Eclipse XDB C_8_ column (150 mm × 4.6 mm; Agilent Technologies, Inc., Santa Clara, CA, USA) maintained at 25 °C, and photo-diode array detector set to monitor 350 and 700 nm was used. The injection volume was 500 µL. The flow was 1 mL/min. The total analysis time was 40 min. Eluent A of the mobile phase was a mixture of MeOH/ACN/0.25 M aqueous pyridine (pH 5.0) in proportion 50/25/25 (*v*/*v*/*v*), and eluent B was a mixture of MeOH/ACN/acetone in proportion 20/60/20 (*v*/*v*/*v*). The linear gradient of solvent A was the following: from 100% in the 1st min to 60% in the 22nd min, from 60% in the 22nd min to 5% in the 38th min, and from 5% in the 38th min to 100% in the 40th min. Analytical data were integrated using ChemStation software version C.01.09 for LC systems (Agilent Technologies, Inc., Santa Clara, CA, USA).

### 4.6. Determination of Monosaccharides

The monosaccharides content was measured spectrophotometrically (Hitachi U-5100 UV-Vis spectrophotometer; Hitachi High-Tech Science Corporation, Tokyo, Japan) using the methods of Nelson [75] and Somogyi [76] with modifications. For this purpose, 0.5 g of *W. arrhiza* powder was extracted in 5 mL of 62.5% (*v*/*v*) MeOH in a water bath (30 min at 60 °C) [77]. The standard sample was 30 mg of glucose dissolved in 62.5% (*v*/*v*) MeOH. The blind sample was distilled water. A 0.5 mL volume of all samples was mixed with 0.5 mL of copper reagent and placed in a boiling water bath for 20 min. Then, 0.5 mL of arsenomolybdate reagent was added, and after 5 min the extract was diluted in 3.5 mL of water and mixed. The absorbance measurement was performed at 540 nm.

### 4.7. Determination of Soluble Proteins

The soluble protein content in *W. arrhiza* was measured spectrophotometrically according to the Bradford [78] method. The basis of this method is the ability to create ionic and hydrophobic bonds between the protein and the Coomassie Brilliant Blue G-250 dye. The Bradford reagent was prepared by dissolving the target dye in 250 mL of 95% (*v*/*v*) EtOH. Next, the obtained mixture was filtered, and then 85% (*w*/*v*) orthophosphate acid was added. The resulting reagent was filled with water to 1000 mL. For the albumin standard preparation, 30 mg of bovine albumin was dissolved in 100 mL of distilled water. For the preparation of the analytic samples, the filtered plants were extracted in 1/4 dilution of the Bradford reagent. The standard sample was albumin with Bradford reagent, while the blind sample was distilled water with Bradford reagent. Then, 3 mL of distilled water was added to all samples. The absorbance was read at 595 nm 60 min after sample preparation.

### 4.8. Determination of Malondialdehyde and H_2_O_2_ Content

Before the level of stress markers was measured, the extract of *W. arrhiza* was prepared. Therefore, 0.1 g of duckweed pellet was suspended in 2 mL of 0.1% (*w*/*v*) trichloroacetic acid (TCA). The obtained mixture was homogenized in a bead mill (50 Hz, 10 min, 4 °C) and centrifuged (2800× *g*, 10 min). The H_2_O_2_ content was determined according to the procedure of Junglee et al. [79] with slight modifications. Thus, 0.5 mL of the resulting supernatant was suspended in 0.5 mL of 10 mM potassium phosphate buffer (pH = 7.0) with 1 mL of 1 M potassium iodide (KI) solution. The standard sample was a 1 mM H_2_O_2_ solution with 10 mM potassium phosphate buffer (pH = 7.0) and 1 M KI. The obtained extracts were left in darkness for 60 min. The level of H_2_O_2_ was determined spectrophotometrically at a wavelength of 390 nm. For MDA analysis, 0.5 mL of supernatant was mixed with 2 mL of 0.5% thiobarbiturate acid (TBA) in 20% TCA. The resulting mixture was incubated in bath water for 20 min at 95 °C. Then, the extract was cooled and centrifuged (2800× *g*, 10 min). The level of MDA was measured spectrophotometrically at a wavelength of 532 nm, and the nonspecific absorption at 600 nm was subtracted. The content of the MDA-TBA complex was calculated using the molar extinction coefficient at 155 mM/cm [80].

### 4.9. Determination of Glutathione and Phytochelatins

For the analysis of the GSH and PCs content, 0.2 g of *W. arrhiza* pellet was placed in an Eppendorf tube containing 1 mL trifluoroacetic acid (TFA) with 6.3 mM diethylenetriaminepentaacetic acid (DTPA) and homogenized in a bead mill (50 Hz, 5 min). The obtained homogenate was centrifuged at 4 °C (2800× *g*, 10 min) for precipitation of protein and cell fragments. For derivatization of the thiol groups, 250 μL of the resulting supernatant was mixed with 450 μL of 200 mM 4-(2-hydroxyethyl)piperazine-1-propanesulphonic acid buffer (HEPPS) with 6.3 mM DTPA and 10 μL of 20 mM monobromobimane (mBBr) solution. The resulting mixture was incubated at 45 °C for 30 min in the dark using the thermomixer. After this, the reaction was stopped by adding 300 μL of 1M methanesulfonic acid solution. Measurement of GSH and PC levels was performed using HPLC. Therefore, 25 μL of each sample was injected into the Cosmosil C_18_-MS-II column (250 mm × 4.6 µm, 5 µm). The oven column temperature was 37 °C. The flow was 0.5 mL/min. Mobile phase A was MeOH, and mobile phase B was aqueous 0.1% TFA. The linear gradient of phase A was the following: 12–25% from 1 to 15 min; 25–35% from 15 to 29 min; 35–50% from 29 to 50 min; 50–100% from 50 to 70 min; and 12% after 70 min. The analysis time was 70 min. The retention times of GSH and PCs were established using their standards. The wavelengths of excitation and emission were 380 and 470 nm, respectively [51,81].

### 4.10. Determination of Antioxidants

Before measuring the antioxidant activity, the enzyme extracts were prepared. Therefore, the *W. arrhiza* pellet was suspended in the reaction mixture containing 0.05 M phosphate buffer (pH = 7.0), 1 mM phenylmethanesulfonylfluoride, 1 mM EDTA, 0.5% (*v*/*v*) Triton X-100, and 2% (*w*/*v*) polyvinylpyrrolidone. The obtained mixture was homogenized in a bead mill (50 Hz, 10 min) and centrifuged (2800× *g*, 10 min, 4 °C).

For determination of CAT activity, 0.1 mL of the enzyme extract was suspended in 0.05 M potassium phosphate buffer (pH = 7.0) with 1 mM H_2_O_2_. The total value of the reaction mixture was 3 mL. The decrease in H_2_O_2_ absorbance at the wavelength of 240 nm for 1 min of reaction was measured spectrophotometrically [82]. A unit (U) of CAT activity was assumed as the amount of enzyme that decomposes 1 μM of H_2_O_2_ per milligram of soluble protein per minute at 25 °C.

One U of GR activity was measured spectrophotometrically from the rate of NADPH oxidation by the decrease in absorbance at 340 nm for 1 min of reaction (extinction coefficient 6.2 mM/cm). Three milliliters of the reaction mixture contained 0.1 mL of enzymatic extract, 0.05 M potassium phosphate buffer (pH = 7.6), 1 mM EDTA, 1 mM NADPH, and 1 mM oxidized glutathione (GSSG). The reaction was started after the addition of NADPH at 25 °C [83].

One U of APX activity was measured spectrophotometrically as the amount of the enzyme that oxidizes 1 μM of ascorbate per milligram of soluble protein per minute at 25 °C. Absorbance measurement was performed at 290 nm for 1 min of reaction (extinction coefficient 2.8 mM/cm). Three milliliters of the reaction mixture consisted of 0.1 mL of enzyme extract, 0.05 M potassium phosphate buffer (pH = 7.0), 0.5 mM ascorbate, and 1 mM H_2_O_2_ [84].

For determination of SOD activity, 0.1 mL of enzymatic extract was mixed with 0.05 M sodium carbonate (pH 10.2), 0.1 mM EDTA, 0.024 mM solution of nitroblue tetrazolium (NBT) 0.03% (*v*/*v*) Triton X-100, and 1 mM hydroxylamine. The total value of the reaction mixture was 3 mL. SOD activity was assayed by measuring its ability to inhibit the photochemical reduction of NBT at 560 nm. One U of SOD activity (per milligram protein) was assumed as the amount that causes a 50% inhibition of the photochemical reduction of NBT [85].

### 4.11. Statistical Analysis

All statistical analyses were performed with Statistica 13 software [86]. Basic descriptive statistics were calculated for the dataset grouped by treatment (*n* = 5, biological replicate). The Shapiro–Wilk test was used to verify the normality of the tested samples. Then, the statistical differences between groups were determined by one-way analysis of variance (ANOVA), followed by honestly significant differences Tukey’s post hoc tests. Differences were considered significant for *p* < 0.05. The results of the ANOVA were shown as plots and tables.

## Figures and Tables

**Table 1:** Intracellular cadmium (Cd) content in *W. arrhiza* (mg/g dry weight) exposed to Cd or Cd with melatonin (MT) after 7 days of cultivation, followed by the content of Cd in medium (mg/L) on the 1st and 7th day of cultivation. The results present the mean (*n* = 5, biological replicate) ± standard deviation. The range in square brackets corresponds to the 95% confidence interval of the means. Means with the same letters are not significantly different (*p* ≥ 0.05) according to Tukey’s post hoc test.

Treatment	Cd Content in *W. arrhiza*	Cd Content in Medium Day 1	Cd Content in Medium Day 7
Control	0	0	0
0.1 µM Cd	0.026 ± 0.002 ^k^ [0.024–0.028]	0.007 ± 0.001 ^g^ [0.006–0.007]	0.005 ± 0.001 ^k^ [0.005–0.006]
1 µM Cd	0.31 ± 0.016 ^k^ [0.292–0.328]	0.101 ± 0.002 ^g^ [0.099–0.103]	0.088 ± 0.004 ^k^ [0.084–0.092]
5 µM Cd	1.655 ± 0.036 ^jk^ [1.614–1.696]	0.652 ± 0.005 ^f^ [0.647–0.657]	0.542 ± 0.004 ^j^ [0.538–0.547]
10 µM Cd	4.088 ± 0.068 ^i^ [4.011–4.165]	1.435 ± 0.008 ^e^ [1.425–1.444]	1.155 ± 0.049 ^i^ [1.099–1.211]
25 µM Cd	10.788 ± 0.168 ^g^ [10.598–10.979]	3.139 ± 0.005 ^d^ [3.133–3.145]	2.572 ± 0.035 ^h^ [2.533–2.612]
50 µM Cd	24.66 ± 1.424 ^e^ [23.049–26.271]	6.079 ± 0.042 ^c^ [6.032–6.126]	5.178 ± 0.011 ^f^ [5.166–5.19]
75 µM Cd	34.784 ± 0.618 ^c^ [34.085–35.483]	8.130 ± 0.008 ^b^ [8.121–8.138]	7.18 ± 0.03 ^d^ [7.146–7.214]
100 µM Cd	42.076 ± 1.695 ^a^ [40.158–43.994]	11.652 ± 0.308 ^a^ [11.303–12.001]	10.299 ± 0.019 ^b^ [10.278–10.32]
0.1 µM Cd + 25 µM MT	0.021 ± 0.002 ^k^ [0.019–0.023]	0.007 ± 0.001 ^g^ [0.006–0.008]	0.007 ± 0.001 ^k^ [0.006–0.007]
1 µM Cd + 25 µM MT	0.28 ± 0.008 ^k^ [0.272–0.289]	0.103 ± 0.004 ^g^ [0.099–0.108]	0.094 ± 0.002 ^k^ [0.092–0.096]
5 µM Cd + 25 µM MT	1.274 ± 0.101 ^k^ [1.159–1.389]	0.658 ± 0.006 ^f^ [0.651–0.666]	0.596 ± 0.057 ^j^ [0.531–0.66]
10µM Cd + 25 µM MT	3.232 ± 0.118 ^ij^ [3.099–3.365]	1.447 ± 0.008 ^e^ [1.438–1.456]	1.225 ± 0.004 ^i^ [1.221–1.229]
25 µM Cd + 25 µM MT	8.783 ± 0.087 ^h^ [8.684–8.882]	3.099 ± 0.02 ^d^ [3.076–3.121]	2.799 ± 0.081 ^g^ [2.707–2.891]
50 µM Cd + 25 µM MT	22.241 ± 0.393 ^f^ [21.796–22.685]	6.082 ± 0.023 ^c^ [6.055–6.108]	5.586 ± 0.09 ^e^ [5.484–5.689]
75 µM Cd + 25 µM MT	32.366 ± 0.752 ^d^ [31.515–33.217]	8.137 ± 0.012 ^b^ [8.124–8.15]	7.548 ± 0.066 ^c^ [7.473–7.622]
100 µM Cd + 25 µM MT	38.342 ± 0.565 ^b^ [37.703–38.981]	11.556 ± 0.25 ^a^ [11.273–11.839]	10.906 ± 0.085 ^a^ [10.81–11.001]

**Table 2:** The endogenous level of melatonin (MT) and its intermediates (ng/g fresh weight) in *W. arrhiza* exposed to cadmium (Cd) or/with MT after 7 days of cultivation. The results present the mean (*n* = 5, biological replicate) ± standard deviation. The range in square brackets corresponds to the 95% confidence interval of the means. Means with the same letters are not significantly different (*p* ≥ 0.05) according to Tukey’s post hoc test.

Treatment	MT	5HT	NAS	Trp	5HTP	TAM	5MT
Control	12.934 ± 1.01 ^o^ [12.053–13.816]	62.23 ± 0.96 ^m^ [61.387–63.067]	1.195 ± 0.06 ^n^ [1.14–1.25]	31.322 ± 1.83 ^n^ [29.717–32.926]	6.523 ± 0.33 ^j^ [6.234–6.811]	1.426 ± 0.02 ^m^ [1.412–1.439]	4.866 ± 0.18 ^k^ [4.704–5.027]
0.1 µM Cd	15.887 ± 1.01 ^o^ [14.999–16.774]	64.69 ± 0.84 ^lm^ [63.954–65.424]	1.337 ± 0.05 ^m^ [1.297–1.377]	35.087 ± 1.67 ^m^ [33.620–36.555]	7.534 ± 0.07 ^ij^ [7.472–7.597]	1.686 ± 0.03 ^l^ [1.663–1.709]	6.027 ± 0.18 ^j^ [5.871–6.182]
1 µM Cd	24.761 ± 1.92 ^n^ [23.075–26.448]	68.74 ± 0.9 ^k^ [67.953–69.531]	1.57 ± 0.03 ^kl^ [1.548–1.593]	46.269 ± 2.08 ^k^ [44.447–48.091]	9.488 ± 0.11 ^gh^ [9.393–9.583]	2.374 ± 0.05 ^k^ [2.329- 2.42]	7.904 ± 0.56 ^i^ [7.413–8.394]
5 µM Cd	32.286 ± 1.24 ^m^ [31.197–33.375]	87.29 ± 1.29 ^i^ [86.164–88.424]	1.674 ± 0.03 ^jk^ [1.645–1.703]	54.031 ± 2.01 ^j^ [52.269–55.794]	12.440 ± 0.06 ^f^ [12.39–12.491]	3.394 ± 0.05 ^i^ [3.352–3.436]	9.37 ± 0.17 ^gh^ [9.222–9.518]
10 µM Cd	42.739 ± 1.46 ^l^ [41.456–44.022]	97.26 ± 1.61 ^g^ [95.85–98.676]	2.087 ± 0.05 ^h^ [2.046–2.129]	72.012 ± 1.63 ^h^ [70.583–73.44]	14.506 ± 0.42 ^e^ [14.141–14.871]	3.834 ± 0.04 ^h^ [3.801–3.867]	11.036 ± 0.49 ^f^ [10.603–11.469]
25 µM Cd	52.582 ± 1.42 ^j^ [51.334–53.829]	119.17 ± 1.17 ^e^ [118.147–120.202]	2.483 ± 0.02 ^f^ [2.464–2.503]	112.670 ± 1.99 ^e^ [110.922–114.417]	16.816 ± 0.45 ^d^ [16.418–17.214]	4.283 ± 0.04 ^fg^ [4.245–4.321]	13.771 ± 0.37 ^d^ [13.447–14.095]
50 µM Cd	62.585 ± 1.4 ^i^ [61.354–63.817]	128.04 ± 1.44 ^c^ [126.776–129.295]	3.154 ± 0.06 ^c^ [3.102–3.205]	117.259 ± 0.73 ^d^ [116.624–117.895]	18.793 ± 0.52 ^c^ [18.338–19.247]	4.784 ± 0.07 ^d^ [4.723–4.846]	15.998 ± 0.33 ^c^ [15.707–16.289]
75 µM Cd	55.594 ± 1.96 ^j^ [53.875–57.312]	123.75 ± 1.3 ^d^ [122.611–124.882]	3.012 ± 0.01 ^d^ [3.007–3.017]	113.294 ± 1.36 ^e^ [112.103–114.486]	16.286 ± 0.81 ^d^ [15.573–16.998]	4.428 ± 0.03 ^ef^ [4.4–4.457]	15.060 ± 0.31 ^c^ [14.786–15.334]
100 µM Cd	48.83 ± 1.61 ^k^ [47.421–50.239]	119.58 ± 2.13 ^e^ [117.718–121.45]	2.935 ± 0.06 ^d^ [2.880–2.991]	109.058 ± 1.11 ^f^ [108.088–110.028]	13.842 ± 1.02 ^e^ [12.950–14.735]	4.245 ± 0.03 ^g^ [4.215–4.275]	13.681 ± 0.95 ^d^ [12.848–14.515]
25 µM MT	148.624 ± 1.57 ^h^ [147.246–150.001]	65.17 ± 1.11 ^lm^ [64.194–66.144]	1.308 ± 0.03 ^m^ [1.286–1.330]	39.542 ± 1.12 ^l^ [38.560–40.523]	7.535 ± 0.25 ^ij^ [7.32–7.75]	1.5 ± 0.01 ^m^ [1.494–1.506]	5.598 ± 0.06 ^jk^ [5.542–5.653]
0.1 µM Cd + 25 µM MT	153.043 ± 1.64 ^g^ [151.602–154.484]	67.59 ± 1.14 ^kl^ [66.595–68.586]	1.497 ± 0.01 ^l^ [1.489–1.506]	41.298 ± 0.84 ^l^ [40.563–42.033]	8.540 ± 0.3 ^hi^ [8.281–8.799]	1.749 ± 0.04 ^l^ [1.715–1.783]	6.516 ± 0.04 ^j^ [6.481–6.552]
1 µM Cd + 25 µM MT	164.182 ± 1.5 ^f^ [162.864–165.501]	76.97 ± 2.01 ^j^ [75.207–78.729]	1.745 ± 0.02 ^j^ [1.724–1.766]	53.779 ± 1.17 ^j^ [52.752–54.805]	10.268 ± 0.28 ^g^ [10.022–10.514]	3.046 ± 0.11 ^j^ [2.952–3.140]	8.270 ± 0.6 ^hi^ [7.747–8.794]
5 µM Cd + 25 µM MT	172.387 ± 0.8 ^e^ [171.686–173.088]	93.96 ± 1.21 ^h^ [92.908–95.021]	1.902 ± 0.02 ^i^ [1.882–1.923]	65.887 ± 0.45 ^i^ [65.494–66.28]	14.429 ± 0.68 ^e^ [13.833–15.024]	3.889 ± 0.08 ^h^ [3.822–3.956]	10.061 ± 0.12 ^fg^ [9.957–10.166]
10 µM Cd + 25 µM MT	181.929 ± 1.03 ^d^ [181.028–182.829]	104.05 ± 1.8 ^f^ [102.474–105.624]	2.353 ± 0.05 ^g^ [2.311–2.395]	80.687 ± 0.83 ^g^ [79.960–81.414]	17.004 ± 0.52 ^d^ [16.546–17.462]	4.498 ± 0.13 ^e^ [4.384–4.611]	12.404 ± 0.42 ^e^ [12.039–12.769]
25 µM Cd + 25 µM MT	202.728 ± 1.66 ^c^ [201.275–204.181]	130.28 ± 1.81 ^bc^ [128.691–131.862]	2.815 ± 0.04 ^e^ [2.784–2.846]	120.683 ± 1.3 ^bc^ [119.546–121.821]	20.347 ± 0.9 ^b^ [19.556–21.138]	4.840 ± 0.09 ^d^ [4.765–4.914]	16.017 ± 0.6 ^c^ [15.491–16.543]
50 µM Cd + 25 µM MT	232.417 ± 1.32 ^a^ [231.262–233.573]	134.98 ± 1.22 ^a^ [133.910–136.049]	3.494 ± 0.03 ^a^ [3.471–3.517]	126.791 ± 0.88 ^a^ [126.020–127.562]	24.211 ± 0.59 ^a^ [23.696–24.726]	5.473 ± 0.11 ^a^ [5.375–5.572]	19.133 ± 0.43 ^a^ [18.755–19.511]
75 µM Cd + 25 µM MT	233.685 ± 1.47 ^a^ [232.399–234.971]	132.33 ± 1.12 ^ab^ [131.346–133.317]	3.324 ± 0.09 ^b^ [3.248–3.4]	123.005 ± 0.96 ^b^ [122.160–123.849]	21.623 ± 0.94 ^b^ [20.801–22.444]	5.304 ± 0.14 ^b^ [5.179–5.429]	18.829 ± 0.82 ^a^ [18.108–19.549]
100 µM Cd + 25 µM MT	223.099 ± 1.52 ^b^ [221.769–224.429]	122.28 ±1.25 ^de^ [121.177–123.375]	3.118 ± 0.08 ^c^ [3.052–3.184]	117.845 ± 1.09 ^cd^ [116.888–118.801]	18.524 ± 0.86 ^c^ [17.771–19.277]	5.118 ± 0.08 ^c^ [5.052–5.185]	17.714 ± 0.71 ^b^ [17.088–18.34]

5HT, serotonin; 5HTP, 5-hydroxytryptophan; 5MT, 5-methoxytryptamine; MT, melatonin; NAS, *N*-acetylserotonin; TAM, tryptamine; Trp, tryptophan.

**Table 3:** The endogenous level of photosynthetic pigments (µg/ g fresh weight) in *W. arrhiza* exposed to cadmium (Cd) or/with melatonin (MT) after 7 days of cultivation. The results present the mean (*n* = 5, biological replicate) ± standard deviation. The range in square brackets corresponds to the 95% confidence interval of the means. Means with the same letters are not significantly different (*p* ≥ 0.05) according to Tukey’s post hoc test.

Treatment	Chlorophyll *a*	Chlorophyll *b*	α-Carotene	β-Carotene	Neoxanthin
Control	162.155 ± 2.69 ^e^ [159.798–164.513]	42.194 ± 2.2 ^ef^ [40.262–44.127]	1.289 ± 0.01 ^e^ [1.281–1.297]	1.78 ± 0.07 ^d^ [1.716–1.844]	0.917 ± 0.04 ^e^ [0.884–0.95]
0.1 µM Cd	159.030 ± 1.13 ^e^ [158.040–160.019]	40.155 ± 0.73 ^fg^ [39.514–40.796]	1.235 ± 0.01 ^e^ [1.223–1.247]	1.701 ± 0.07 ^de^ [1.64–1.761]	0.906 ± 0.02 ^e^ [0.89–0.922]
1 µM Cd	153.193 ± 1.15 ^f^ [152.186–154.199]	39.324 ± 0.56 ^g^ [38.837–39.811]	1.095 ± 0.03 ^fg^ [1.072–1.118]	1.619 ± 0.03 ^ef^ [1.596–1.642]	0.864 ± 0.02 ^e^ [0.846–0.882]
5 µM Cd	146.853 ± 1.09 ^g^ [145.896–147.809]	36.140 ± 0.76 ^h^ [35.472–36.809]	1.04 ± 0.01 ^g^ [1.029–1.050]	1.531 ± 0.05 ^f^ [1.486–1.576]	0.786 ± 0.01 ^f^ [0.779–0.792]
10 µM Cd	140.7 ± 0.97 ^h^ [139.852–141.548]	33.991 ± 0.38 ^hi^ [33.659–34.323]	0.944 ± 0.02 ^h^ [0.929–0.959]	1.411 ± 0.03 ^g^ [1.386–1.436]	0.739 ± 0.02 ^f^ [0.724–0.753]
25 µM Cd	127.306 ± 0.94 ^i^ [126.486–128.126]	30.056 ± 0.53 ^jk^ [29.593–30.519]	0.809 ± 0.01 ^j^ [0.796–0.822]	1.235 ± 0.07 ^hi^ [1.174–1.295]	0.652 ± 0.01 ^g^ [0.645–0.66]
50 µM Cd	92.663 ± 1.36 ^l^ [91.467–93.858]	26.034 ± 0.41 ^l^ [25.679–26.389]	0.724 ± 0.02 ^k^ [0.71–0.739]	1.076 ± 0.07 ^j^ [1.01–1.141]	0.569 ± 0.02 ^ij^ [0.55–0.588]
75 µM Cd	78.585 ± 1.21 ^m^ [77.524–79.646]	21.454 ± 0.90 ^m^ [20.667–22.240]	0.633 ± 0.01 ^l^ [0.625–0.641]	0.949 ± 0.02 ^k^ [0.932–0.966]	0.514 ± 0.01 ^jk^ [0.501–0.526]
100 µM Cd	65.017 ± 1.83 ^n^ [63.416–66.619]	18.041 ± 0.82 ^n^ [17.324–18.758]	0.591 ± 0.02 ^l^ [0.576–0.606]	0.895 ± 0.02 ^k^ [0.878–0.913]	0.473 ± 0.01 ^k^ [0.462–0.484]
25 µM MT	237.154 ± 1.76 ^a^ [235.61–238.698]	67.216 ± 1.26 ^a^ [66.11–68.323]	1.794 ± 0.02 ^a^ [1.773–1.814]	2.911 ± 0.04 ^a^ [2.872–2.949]	1.442 ± 0.03 ^a^ [1.411–1.472]
0.1 µM Cd + 25 µM MT	228.022 ± 1.53 ^b^ [226.678–229.365]	65.336 ± 0.95 ^ab^ [64.504–66.167]	1.722 ± 0.07 ^b^ [1.659–1.785]	2.807 ± 0.03 ^a^ [2.784–2.831]	1.389 ± 0.04 ^a^ [1.353–1.424]
1 µM Cd + 25 µM MT	219.783 ± 0.92 ^c^ [218.977–220.588]	63.692 ± 0.55 ^b^ [63.207–64.176]	1.7 ± 0.01 ^bc^ [1.687–1.713]	2.596 ± 0.06 ^b^ [2.545–2.647]	1.286 ± 0.02 ^b^ [1.265–1.308]
5 µM Cd + 25 µM MT	173.649 ± 1.68 ^d^ [172.181–175.118]	60.752 ± 0.85 ^c^ [60.006–61.497]	1.651 ± 0.05 ^c^ [1.603–1.699]	2.324 ± 0.05 ^c^ [2.28–2.369]	1.222 ± 0.03 ^c^ [1.195–1.248]
10 µM Cd + 25 µM MT	160.743 ± 1.51 ^e^ [159.42–162.067]	48.853 ± 0.90 ^d^ [48.068–49.638]	1.415 ± 0.05 ^d^ [1.367–1.463]	1.705 ± 0.03 ^de^ [1.68–1.730]	1.076 ± 0.04 ^d^ [1.044–1.108]
25 µM Cd + 25 µM MT	147.226 ± 2.71 ^g^ [144.847–149.604]	42.715 ± 0.88 ^e^ [41.946–43.483]	1.121 ± 0.04 ^f^ [1.086–1.156]	1.414 ± 0.04 ^g^ [1.379–1.450]	0.874 ± 0.03 ^e^ [0.844–0.903]
50 µM Cd + 25 µM MT	122.811 ± 1.92 ^j^ [121.133–124.490]	35.349 ± 0.93 ^h^ [34.534–36.164]	0.897 ± 0.02 ^hi^ [0.88–0.914]	1.292 ± 0.04 ^h^ [1.26–1.325]	0.667 ± 0.03 ^g^ [0.639–0.695]
75 µM Cd + 25 µM MT	104.141 ± 0.75 ^k^ [103.483–104.799]	32.233 ± 1.12 ^ij^ [31.255–33.210]	0.838 ± 0.01 ^ij^ [0.829–0.847]	1.180 ± 0.03 ^ij^ [1.156–1.205]	0.629 ± 0.02 ^gh^ [0.613–0.645]
100 µM Cd + 25 µM MT	93.883 ± 2.69 ^l^ [91.526–96.240]	29.073 ± 1.0 ^k^ [28.201–29.945]	0.791 ± 0.02 ^jk^ [0.774–0.808]	1.155 ± 0.02 ^ij^ [1.141–1.169]	0.586 ± 0.02 ^hi^ [0.57–0.603]
	**Violaxanthin**	**Astaxanthin**	**Zeaxanthin**	**Cryptoxanthin**	**Lutein**
Control	0.7 ± 0.01 ^ef^ [0.69–0.711]	0.352 ± 0.02 ^e^ [0.331–0.373]	3.208 ± 0.06 ^f^ [3.158–3.258]	4.237 ± 0.08 ^de^ [4.168–4.307]	0.473 ± 0.01 ^d^ [0.465–0.481]
0.1 µM Cd	0.653 ± 0.03 ^fg^ [0.628–0.678]	0.328 ± 0.01 ^ef^ [0.32–0.337]	3.143 ± 0.01 ^fg^ [3.135–3.151]	4.141 ± 0.05 ^ef^ [4.094–4.188]	0.422 ± 0.01 ^e^ [0.411–0.434]
1 µM Cd	0.625 ± 0.01 ^g^ [0.616–0.635]	0.317 ± 0.01 ^ef^ [0.31–0.324]	3.079 ± 0.01 ^g^ [3.068–3.089]	4.061 ± 0.02 ^fg^ [4.04–4.082]	0.392 ± 0.01 ^ef^ [0.381–0.403]
5 µM Cd	0.554 ± 0.01 ^hi^ [0.542–0.565]	0.292 ± 0.01 ^fg^ [0.282–0.302]	2.904 ± 0.02 ^h^ [2.884–2.924]	3.880 ± 0.06 ^h^ [3.826–3.934]	0.371 ± 0.01 ^fg^ [0.364–0.378]
10 µM Cd	0.526 ± 0.01 ^hij^ [0.521–0.531]	0.265 ± 0.01 ^gh^ [0.256–0.274]	2.770 ± 0.02 ^i^ [2.749–2.792]	3.521 ± 0.02 ^i^ [3.502–3.540]	0.349 ± 0.02 ^gh^ [0.334–0.363]
25 µM Cd	0.486 ± 0.01 ^jk^ [0.476–0.496]	0.238 ± 0.01 ^hi^ [0.23–0.246]	2.512 ± 0.02 ^k^ [2.496–2.529]	3.362 ± 0.02 ^j^ [3.344–3.381]	0.309 ± 0.02 ^i^ [0.296–0.323]
50 µM Cd	0.464 ± 0.01 ^k^ [0.454–0.474]	0.207 ± 0.01 ^ij^ [0.197–0.216]	2.236 ± 0.04 ^l^ [2.205–2.267]	3.001 ± 0.04 ^l^ [2.970–3.032]	0.276 ± 0.01 ^jk^ [0.265–0.287]
75 µM Cd	0.397 ± 0.02 ^l^ [0.383–0.412]	0.182 ± 0.01 ^jk^ [0.175–0.188]	1.953 ± 0.04 ^m^ [1.922–1.984]	2.790 ± 0.03 ^m^ [2.761–2.820]	0.254 ± 0.01 ^kl^ [0.249–0.258]
100 µM Cd	0.356 ± 0.02 ^l^ [0.342–0.371]	0.147 ± 0.01 ^k^ [0.142–0.152]	1.853 ± 0.05 ^m^ [1.81–1.896]	2.634 ± 0.05 ^n^ [2.593–2.674]	0.242 ± 0.01 ^l^ [0.235–0.249]
25 µM MT	1.146 ± 0.04 ^a^ [1.109–1.183]	0.622 ± 0.03 ^a^ [0.593–0.650]	4.693 ± 0.03 ^a^ [4.663–4.723]	6.490 ± 0.03 ^a^ [6.465–6.515]	0.772 ± 0.01 ^a^ [0.762–0.782]
0.1 µM Cd + 25 µM MT	1.077 ± 0.03 ^b^ [1.049–1.105]	0.558 ± 0.02 ^b^ [0.540–0.576]	4.577 ± 0.09 ^b^ [4.494–4.660]	6.389 ± 0.07 ^a^ [6.325–6.453]	0.736 ± 0.03 ^b^ [0.711–0.760]
1 µM Cd + 25 µM MT	0.966 ± 0.02 ^c^ [0.945–0.987]	0.472 ± 0.02 ^c^ [0.458–0.486]	4.442 ± 0.04 ^c^ [4.403–4.481]	5.95 ± 0.07 ^b^ [5.886–6.013]	0.605 ± 0.02 ^c^ [0.589–0.620]
5 µM Cd + 25 µM MT	0.777 ± 0.02 ^d^ [0.755–0.798]	0.395 ± 0.02 ^d^ [0.377–0.412]	4.256 ± 0.04 ^d^ [4.221–4.292]	5.422 ± 0.12 ^c^ [5.315–5.529]	0.464 ± 0.02 ^d^ [0.447–0.480]
10 µM Cd + 25 µM MT	0.702 ± 0.03 ^e^ [0.677–0.727]	0.346 ± 0.02 ^e^ [0.33–0.361]	3.764 ± 0.04 ^e^ [3.73–3.799]	4.325 ± 0.08 ^d^ [4.257–4.392]	0.405 ± 0.02 ^e^ [0.391–0.419]
25 µM Cd + 25 µM MT	0.658 ± 0.02 ^efg^ [0.641–0.676]	0.325 ± 0.01 ^ef^ [0.315–0.335]	3.088 ± 0.03 ^g^ [3.06–3.116]	3.941 ± 0.04 ^gh^ [3.908–3.973]	0.363 ± 0.02 ^fg^ [0.349–0.377]
50 µM Cd + 25 µM MT	0.57 ± 0.02 ^h^ [0.551–0.589]	0.264 ± 0.02 ^gh^ [0.249–0.279]	2.645 ± 0.06 ^j^ [2.595–2.695]	3.387 ± 0.05 ^j^ [3.347–3.427]	0.347 ± 0.01 ^gh^ [0.339–0.354]
75 µM Cd + 25 µM MT	0.561 ± 0.02 ^hi^ [0.546–0.576]	0.248 ± 0.01 ^h^ [0.236–0.261]	2.336 ± 0.1 ^l^ [2.253–2.42]	3.336 ± 0.03 ^j^ [3.309–3.363]	0.327 ± 0.01 ^hi^ [0.323–0.332]
100 µM Cd + 25 µM MT	0.516 ± 0.01 ^ij^ [0.504–0.529]	0.21 ± 0.02 ^ij^ [0.195–0.226]	2.230 ± 0.03 ^l^ [2.203–2.256]	3.177 ± 0.03 ^k^ [3.154–3.201]	0.297 ± 0.02 ^ij^ [0.284–0.311]

**Table 4:** Chromatographic properties of melatonin and its intermediates in positive ionization scan mode of ESI-LC-MS.

Compound	Scan Mode (ESI)	Precursor *m*/*z*	Product *m*/*z*	CE	Retention Time
Melatonin	+	233.0	174.0	−16	7.380
Serotonin	+	176.85	160.15	−14	1.864
*N*-acetylserotonin	+	219.0	160.25	−18	4.358
Tryptophan	+	204.9	188.15	−22	2.997
5-hydroxytryptophan	+	220.9	204.0	−15	3.093
Tryptamine	+	161.0	144.2	−15	2.242
5-metoxytryptamine	+	190.9	174.15	−13	2.017

## Data Availability

Data are contained within the current article and supplementary material.

## Supplementary Materials

The following are available online at https://www.mdpi.com/article/10.3390/ijms24021178/s1.
