# Supplementary material for: Melatonin Involved in Protective Effects against Cadmium Stress in Wolffia arrhiza
Source: Int J Mol Sci. 2023 Jan 7;24(2):1178. doi: 10.3390/ijms24021178 (PMC9867261; doi:10.3390/ijms24021178)
Supplement: Supplementary file 1 [file ijms-24-01178-s001.zip › ijms-2143433-supplementary.pdf]

International Journal of
Molecular Sciences

melatonin

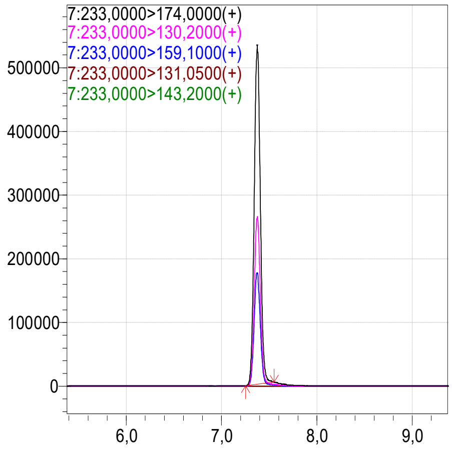

serotonin

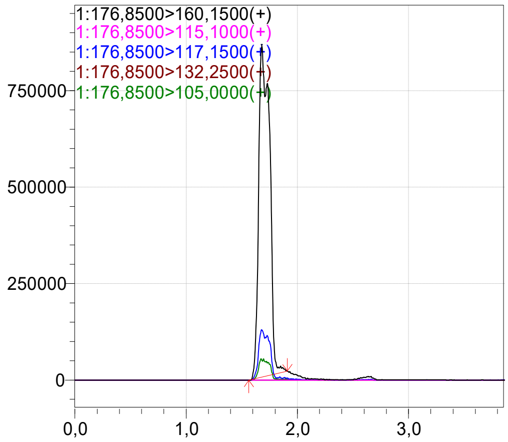

***N*-acetylserotonin**

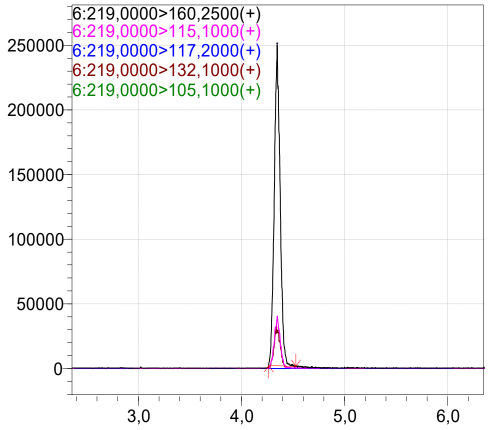

tryptophan

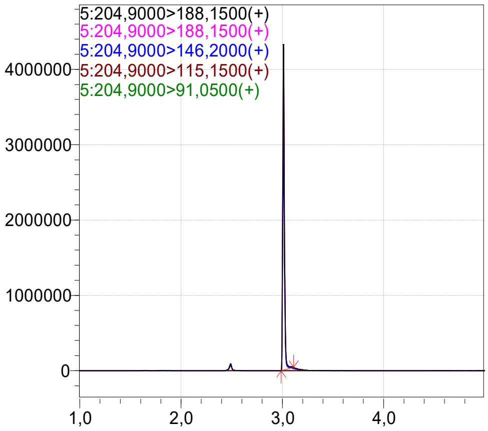

5-hydroxytryptophan

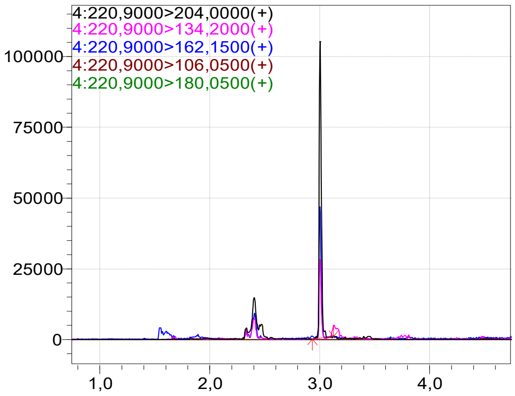

tryptamine

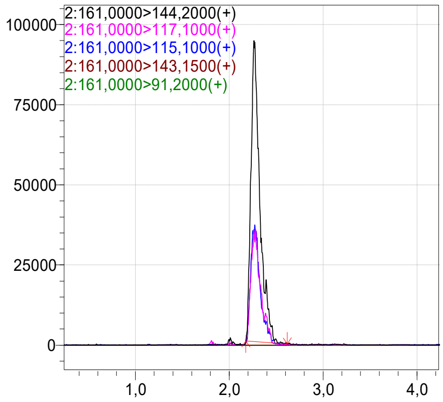

5-metoxytryptamine

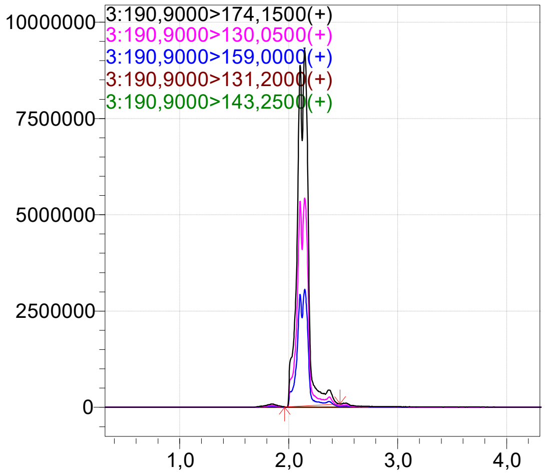

Figure S1. The chromatograms of the detected compounds in *Wolffia arrhiza*, i.e., melatonin and its intermediates: serotonin, *N*-acetylserotonin, tryptophan, 5-hydroxytryptophan, tryptamine, and 5-metoxytryptamine.
